# Economic evaluation of ivabradine in the treatment of chronic heart failure in Greece

**DOI:** 10.1186/s12913-014-0631-0

**Published:** 2014-12-11

**Authors:** Georgia Kourlaba, John Parissis, Apostolos Karavidas, Alexandra Beletsi, Charalambos Milonas, Neil Branscombe, Nikos Maniadakis

**Affiliations:** The Stavros Niarchos Foundation-Collaborative Center for Clinical Epidemiology and Outcomes Research (CLEO), National and Kapodistrian University of Athens, School of Medicine, Athens, Greece; Department of Cardiology, Attikon University Hospital, Athens, Greece; Department of Cardiology, Athens General Hospital “Georgios Genimmatas”, Athens, Greece; Servier Hellas Pharmaceuticals Ltd, Athens, Greece; Department of Health Services Organization & Management, National School of Public Health, Athens, Greece; Servier Laboratories Ltd, Suresnes, France

**Keywords:** Cost-effectiveness, Heart failure, Ivabradine, Cost-utility

## Abstract

**Background:**

The objective of our study was to assess the cost-effectiveness of ivabradine plus standard care (SoC) in chronic heart failure (CHF) patients with sinus rhythm and a baseline heart rate ≥ 75 b.p.m. in Greece, in comparison with current SoC alone.

**Methods:**

An existing cost-effectiveness model consisting of two health states, was adapted to the Greek health care setting. All clinical inputs of the model (i.e. mortality rates, hospitalization rates, NYHA class distribution and utility values) were estimated from SHIFT trial data. All costing data used in the model reflects the year 2013 (in €). An incremental cost effectiveness ratio (ICER) per quality-adjusted life year (QALY) gained was calculated. Deterministic and probabilistic sensitivity analyses (PSA) were conducted. The horizon of analysis was over patient life time and both cost and outcomes were discounted at 3.5% per year. The analysis was conducted from a Greek third party-payer perspective.

**Results:**

The Markov analysis revealed that the discounted quality-adjusted survival was 4.27 and 3.99 QALYs in the ivabradine plus SoC and SoC alone treatment arms, respectively. The cumulative lifetime total cost per patient was €8,665 and €5,873, for ivabradine plus SoC and SoC alone, respectively. The ICER for ivabradine plus SoC versus SoC alone was estimated as €9,986 per QALY gained. The PSA showed that the likelihood of ivabradine plus SoC being cost-effective at a threshold of €36,000/QALY was found to be 95%.

**Conclusions:**

Ivabradine plus SoC may be regarded as a cost-effective option for the treatment in CHF patients in Greece.

**Electronic supplementary material:**

The online version of this article (doi:10.1186/s12913-014-0631-0) contains supplementary material, which is available to authorized users.

## Background

Heart failure (HF) is a major public health concern worldwide [1]. Almost 1–2% of the population in European countries suffers from HF, with the prevalence rising to ≥ 10% among the population aged ≥ 70 years [2]. Moreover, HF has a poor prognosis as 40% of patients die within a year from the diagnosis date but thereafter the mortality is less than 10% per year [3]. Prognosis and patient management are correlated to the severity of heart disease. The main tool used to estimate the heart disease severity is the New York Heart Association (NYHA) classification [4]. Moreover, health-related quality of life (HR-QoL) of HF patients is reduced by the physical, social and emotional limitations imposed by the disease. These symptoms may be caused by the disease itself, by co-morbidities, or can result side effects of treatments [5]. In addition to its substantial effect on morbidity and mortality, HF is one of the most costly chronic diseases in developed countries. Costs associated with HF constitute 1-2% of all healthcare expenditure [6].

Current treatments aim to relieve and stabilize symptoms and prolong survival by stopping, stabilizing or reversing the progression of HF [7]. Standard pharmacological treatment includes beta-blockers, angiotensin-converting-enzyme (ACE) inhibitors and/or angiotensin receptors blockers (ARBs), aldosterone antagonists and diuretics [5]. Ivabradine (Procoralan®) is a new therapeutic option for patients with chronic heart failure (CHF) in sinus rhythm. Ivabradine is a pure heart rate lowering agent, and represents the first in a new class of agents acting by selective and specific inhibition of the cardiac pacemaker *I*_f_ current that controls the spontaneous diastolic depolarisation in the sinus node and regulates heart rate. The cardiac effects are specific to the sinus node with no effect on intra-atrial, atrioventricular or intraventricular conduction times, nor on myocardial contractility or ventricular repolarization [8].

The clinical effect of ivabradine plus standard care (SoC) versus placebo on top of SoC has been evaluated through a Phase III international, multicentre randomised controlled trial (SHIFT trial) [9]. The primary endpoint in SHIFT was a composite of cardiovascular (CV) death or hospitalization for worsening HF. This study showed that ivabradine plus SoC was associated with fewer hospital admissions for worsening HF (first event hazard ratio (HR): 0.74; 95% Confidence Interval (CI): 0.66-0.83, p < 0.0001) and lower mortality rates due to HF (HR: 0.74; 95% CI: 0.58-0.94, p = 0.014). Moreover, SHIFT indicated that ivabradine plus SoC was associated with significantly fewer serious adverse events [9].

While the use of ivabradine for the treatment of patients with CHF may be considered to be an effective option, it may impose additional costs to the third party payers and society. The recent climate of the major financial crisis has resulted in strong health care budgetary constraints. This imposes the need to use treatments which are not only clinically effective but also economically efficient, in order to maximize the value for money spent in health care. Simple cross-therapy and cross-country price comparisons are misleading and are not sufficient to determine policies on whether or not (and how) certain treatments should be used. Total treatment cost should be considered and weighed up against the health benefit of a new treatment in relation to existing ones. This need led to the use of economic assessment of technologies used in health care delivery, in the context of which the cost-effectiveness ratio of new treatments is often assessed in comparison to that of existing alternatives. Clinical trials rarely collect sufficient data on treatment costs and consequences for rigorous economic assessment. Thus, mathematical modeling is required to support decision making [10].

Therefore, the purpose of the present study was to conduct an economic evaluation comparing ivabradine plus SoC versus SoC alone in patients with sinus rhythm with NYHA class II-IV CHF and a baseline heart rate ≥ 75 b.p.m in Greece.

## Methods

In the present study, an existing Markov model developed for the Health Technology Assessment of ivabradine by National Institute for Health and Care Excellence (NICE), [11] was adapted to the Greek health care setting. The model has been based on the results of the SHIFT trial [9], taking a third party-payer perspective. Costs and outcomes that occur beyond one year are discounted at a 3.5% annual rate, consistent with the NICE recommendations [12]. As this study is an economic evaluation analysis and does not involve human subjects no ethics approval issues arise. Input data including human material or human data were derived from other published studies performed with the approval of an appropriate ethics committee.

### Model structure

**Figure 1:**
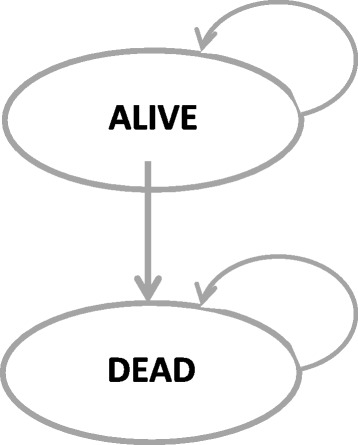
**Structure of the model.**

A simple two state Markov cohort model has been used in the analysis (health states: alive, dead) (Figure 1). Patients enter the model in the “alive” health state and are treated with ivabradine plus SoC or SoC alone. Then, in each subsequent one month cycle during their life span, patients can either remain alive or die from a CV cause (HF and non-HF CV cause) or non-CV cause.

Alive patients are subdivided into NYHA classes (I, II, III and IV) taking into consideration the SHIFT trial data. The proportion of patients in each NYHA class is modeled to change over time. These NYHA states have been included to capture the quality of life (QoL) implications of HF. The rate of hospitalization was also considered in the model but, as with NYHA class, not as separate health states. The hospitalization rates were indirectly modeled to take into account the associated resource use and transient reductions in QoL. A series of multivariable risk equations have been used to predict the risk of mortality, hospitalisation and patient QoL according to treatment allocation and a range of patient baseline characteristics selected on the basis of the SHIFT study protocol, a previous HF risk equation and clinical advice, as described in detail below.

The key assumptions considered in the model are briefly presented in Table 1 and described below in detail.

**Table 1:** **Summary of key assumptions considered in the cost-effectiveness model**

**Parameter**	**Key assumptions**
**Model structure**	Two-stage Markov cohort model
CE model time horizon	Lifetime
Model cycle	Monthly
CV mortality for SoC	SHIFT data by applying Gompertz survival model
HR for CV mortality of ivabradine vs SoC	0.90 (0.80 – 1.03) based on SHIFT data
Rate hospitalization	SHIFT data by applying Poisson model
Rate ratio for hospitalization of ivabradine vs SoC	0.83 (0.78 – 0.93) based on SHIFT data
Length of hospitalization	Local data (based on experts’ opinion)
Utility data	SHIFT data by applying mixed regression model
NYHA data	SHIFT data by applying adjusted ordered logistic regression
Ivabradine treatment effect	Cardiovascular endpoint
Ivabradine use (years)	Lifelong
Cost discount rate per annum	3.5%
Effects discount rate per annum	3.5%
Resource utilization and unit costs	Local data (government gazette and experts’ opinion)
Outcome mesaures	QALYs, LYs, ICER per QALY and per LY gained, lifetime total cost

CE: cost-effectiveness; HR: hazard ratio; QALYs: quality-adjusted life years; LYs: life years; ICER: incremental cost effectiveness ratio; CV: cardiovascular; NYHA: New York Heart Association.

### Comparators/schedules

In the present analysis, ivabradine plus SoC therapy is compared to SoC alone. SoC is considered equivalent across treatment groups. In particular, the SoC drugs included in the analysis reflect those drugs currently recommended in ESC guidelines (beta-blockers, ACE inhibitor, diuretics, aldosterone antagonists, ARBs, cardiac glycosides).

### Model inputs

#### Mortality data

In the base case analysis, CV and non-CV mortality was considered separately. The risk of CV mortality in patients who do not receive ivabradine was estimated based on SHIFT SoC data, by using multivariable risk equations for both the within-trial (29 months) and post-trial extrapolated period. In the base case analysis, the CV mortality risk for patients who received SoC alone was estimated by applying a parametric survival regression model on the mortality data of the full SHIFT cohort. The Gompertz distribution was found to best fit the observed mortality data based on statistical evidence (AIC and BIC criteria), a visual review of Kaplan-Meier survival plots versus predicted curves and the plausibility of predicted survival in the extrapolated, post-trial period [11]. Survival models based on the exponential and Weibull parametric distributions, the next best fitting distributions, and Kaplan Meier data, were included in the model as part of the sensitivity analysis [11].

The CV mortality risk equation was estimated adjusting for a series of baseline patient characteristics. The purpose of including these covariates was to generate different estimates of mortality, depending on the baseline characteristics of populations. The covariates considered for the analysis were patient baseline characteristics; baseline use of heart failure medications; prior use of other cardiac therapies; medical history: prior event; Patient biological characteristics.

The treatment effect on CV mortality for ivabradine plus SoC was estimated by applying a relevant treatment effect (HR) obtained from the parametric model to the underlying mortality risk associated with the SoC group. Specifically, the HR of 0.90 obtained from the Gompertz model was applied.

A separate sensitivity analysis considering only the cause-specific HF mortality endpoint was also considered (with non-HF CV death modeled equivalent to SoC) [11].

The non-CV mortality rate was modeled to be equivalent for ivabradine plus SoC and SoC alone. This rate was estimated by using the age and sex adjusted Greek mortality rates based on the latest data of National Statistics Service (www.statistics.gr).

#### NYHA class

As mentioned above, the proportion of patients in each NYHA class was considered in order to capture the QoL of HF patients. In the base case analysis, the distribution of patients in each NYHA class was estimated by applying a generalized ordered logistic regression model (a proportional odds model) on the SHIFT data. The regression model was designed to predict a time adjusted NYHA distribution for each treatment arm, separately. Because of the lack of any evidence to predict the distribution of patients by NYHA class beyond the SHIFT trial period, we assumed that the proportions remained fixed after the trial period. This proportion was set equal to that observed in the trial at 29 months (LoCF). This approach was considered more conservative than the extrapolation of SHIFT data using the ordered logistic regression [11].

#### Hospitalizations

As with the NYHA class, the hospitalization rates related to HF, other CV causes and all causes for both arms under study were considered in the model to capture the appropriate resource use and the impact on patient’s QoL. These rates were estimated through Poisson regression models applied on the SHIFT data. In the base case analysis, the all-cause hospital admissions were considered. The treatment effect of ivabradine on the rate of admissions to hospital was estimated using a rate ratio of 0.83 derived from the Poisson regression model. The treatment effect was modeled on the all-cause admission because CV and HF admissions were assumed to be implicitly captured in all-cause admission. Admission to hospital after the trial was modeled to be equivalent to the within-trial period and assumed to occur at a constant rate throughout the model irrespective of the ageing population [11].

#### Hospitalization length of stay and type of ward

The length of stay (LoS) associated with hospital admission was estimated using external data based on experts clinical advice. The average LoS was varied according to diagnosis on hospital admission (i.e. HF, other CV diagnosis and non-CV diagnosis). In the base case model, an average LoS of 5 days for all-cause hospitalizations were applied according to data obtained from ministry of health. The average LoS for HF and other CV diagnosis was set at 7 and 4 days, respectively, based on local expert’s advice. Moreover, the experts reported that only 30% of patients with HF spend 3 days in intensive care units (ICUs) and the remaining 4 days in cardiac units, while 70% of patients are hospitalized only in cardiac units.

#### Utilities

Utility values describe the health-related QoL associated with different health states on a scale of zero to one, where zero is equivalent to death and one represents best imaginable health. Due to lack of local utility values for patients with HF, the utility values taken from SHIFT-PRO study in which health-related QoL was captured with EQ-5D questionnaire were considered in the model (Table 2). EQ-5D scores were derived using UK population tariff values [13]. A mixed regression model (designed for longitudinal datasets with repeated observations within respondents) was used to analyze SHIFT EQ-5D scores. The clinical variables considered to potentially predict patient QoL were consistent with those considered in the CV and hospitalization risk equations plus two additional time-varying variables – hospitalization within a 60 day time interval (EQ-5D visit date ±30 days) and NYHA class [11].

**Table 2:** **Utility values and costing data considered in the model**

**Parameters**	**Values**	**Source**
**Utility values**		
No hospitalization		SHIFT trial
NYHA I	0.82	
NYHA II	0.74	
NYHA III	0.64	
NYHA IV	0.46	
Hospitalization		SHIFT trial
NYHA I	−0.04	
NYHA II	−0.07	
NYHA III	−0.10	
NYHA IV	−0.29	
**Drug costs per month**		price bulletin issued by the Greek Ministry of Health [15], law 4052/2012, Government Gazette
Standard care	€35.26	
Ivabradine	€40.52	
**Other therapy related costs**		
ECG	€3.44	
**Hospitalization cost (cost per diem)**		Government Gazzette, Ministerial Desicion 104494, 26/9/2011
Intensive Care Unit	€200	
Cardiac Units	€110	
**Other resource use**		Government Gazzette [16] and local experts’ opinion
On-going HF management costs	€26.05	

HF: Heart Failure; ECG: electrocardiographic changes; NYHA: New York Heart Association.

### Costing methods

The present economic evaluation was conducted by the third-party-payer perspective and as such only health care costs reimbursed by the payer were considered in the analysis. Any other cost, such as the costs related to the central Government budget to cover personnel salaries or patients copayments, was not considered. In particular, hospitalization, medication and HF management costs were considered in the model. All costs (in €) reflect the year 2013.

To estimate the total hospitalization cost, the cost per day for each ward type (i.e. intensive care unit (ICU), general medical, cardiac and rehabilitation) was multiplied by the LoS and the hospitalization rate (described above). The LoS in each ward type as well as the probability of being hospitalized to a cardiac unit or ICU was obtained from local experts. Reimbursement unit costs for bed-day were obtained from the Diagnostic Related Groups (DRGs) tariffs issued by the Greek Ministry of Health [14].

The total monthly cost of ivabradine has been estimated by considering the proportion of patients using 2.5 mg (10%), 5 mg (70%) and 7.5 mg (20%) in Greece (based on experts advice) and the current price of ivabradine as it was obtained from the latest (February 2013) price bulletin issued by the Greek Ministry of Health [15] (Table 2). The price of 2.5 mg was assumed to reflect a half dose of the 5 mg cost, and it is consistent with clinical practice (i.e. the scored tablets may be halved).

The total monthly cost of SoC has been modeled using the overall proportions of patients using each SoC therapy based on local experts opinion, the mean daily dose for each drug as it was reported by local experts and the relevant drug prices (calculated as the cost per mg) (Additional file 1, Table 2). Note that the proportions and daily doses reported by the local experts where in line with those taken by patients in SHIFT study. In case that more than one drug was reported from the experts for a particular SoC therapy group, the weighted cost was considered in the model (Additional file 2). All drug prices were obtained from the latest (February 2013) price bulletin issued by the Greek Ministry of Health [15].

The analysis was undertaken from a payer perspective, hence drug cost was based on the retail product price minus the patient copayment (i.e. reimbursed cost). Moreover, cost is net of two different rebates paid by manufacturers (law 4052/2012, Government Gazette). In particular, a rebate of 9% on the ex factory price is applied as a return for products to be included into the positive list (i.e. a list of reimbursed products). Additionally, a volume rebate, ranging from 0 to 8%, is also applied on the ex factory price based on the quarterly sales of each product. In the present analysis a 5% is assumed based on a realistic assumption about possible sales.

An additional one-off cost was also included for ivabradine therapy titration. Based on local experts advice, dosage titration, in Greece, takes place in a routine visit at the outpatient department of hospital as soon as an ECG has been undertaken. However, social security funds do not reimburse the outpatient visit and as a resultthe dosage titration cost for ivabradine group was considered to be equal to the cost of ECG as it was obtained from the Government Gazette (FEK B’ 3054/2012).

The other modeled resource use for SoC included the on-going HF management (physician visits, outpatient procedures, tests). The number of visits, the laboratory tests (blood and biochemical tests) and cardiological tests (e.g. echo, MRI etc.) required were retrieved from local experts. The health care utilization and costs for managing HF were obtained from Government Gazette [16] and local experts (Table 2).

### Data analysis

The base case approach and data were used to get mean estimates of life time costs, life years (LYs) and quality-adjusted life years (QALYs) for each comparator. When new options like ivabradine are more effective (i.e. higher QALY) and less costly than comparators, they are considered as “dominant” treatments. In cases where new options are less effective and more costly they are considered as “dominated” by the alternatives. In cases where they are associated with higher QALY and higher cost they are considered as cost-effective only when the incremental cost-effectiveness ratio (ICER) is lower than a specific predetermined threshold [17-19]. For a treatment to be considered cost-effective a threshold of €36,000 per QALY was used in the current analysis. This is based on the WHO guidelines that states that a treatment should be considered cost-effective if the ICER is between 1 or 3 times the gross domestic product (GDP) per capita of that country and a treatment is considered highly cost effective at less than 1 times the GDP per capita [20]. The GDP per capita in Greece was estimated at €18,000 taken from the IMF estimation of GDP per capita using current prices [21].

The majority of input data used in the current model are subjected to variation. Therefore, a probabilistic sensitivity analysis (PSA) was conducted to quantify uncertainty. Multivariate regression functions generated using SHIFT individual patient data have been entered in the model along with a Cholesky decomposition to account for correlated parameters. Monte Carlo simulation has been used to generate the resulting joint distributions of total costs and QALYs in the model [22]. The model outputs have also been expressed in terms of ‘decision uncertainty’ using cost-effectiveness acceptability curves (CEACs) which show the probability of each therapy being cost-effective given a particular threshold value for cost-effectiveness [23].

Deterministic sensitivity analysis was performed to identify the parameters and structural assumptions to which the model was most sensitive. The upper and lower bounds of parameters were defined by 95% CIs where possible or plausible variation around the base case values.

Subgroup analyses have been performed for subgroup populations identified from the clinical study protocol. These included age (< or ≥75 years old); HF duration (categorized by quartile cut points); NYHA class; Left Ventricular Ejection Fraction (categorized by quartile cut points); prior ischaemia; prior diabetes and β-blocker use.

All statistical calculations performed using Microsoft Excel 2010.

## Results

### Deterministic results

In the base case analysis, the Markov model revealed that the discounted survival was higher in ivabradine plus SoC arm compared to the SoC alone by 0.25 LYs, while the corresponding discounted QALYs were increased by 0.28 QALYs.

Moreover, it was found that ivabradine plus SoC was the most costly treatment regimen versus SoC alone, in the lifetime horizon (€8,665 versus €5,873, respectively). Among the cost categories considered, drug acquisition costs account for 61% and 40% of total costs in ivabradine plus SoC and SoC alone, respectively. Following drug acquisition cost, hospitalization cost accounts for 21% and 30% of the total costs of ivabradine and SoC, respectively. Finally, follow up costs made up approximately 30% of the total cost for SoC and 17% for ivabradine. Under the base case assumptions, incremental analysis showed that ivabradine plus SoC was more effective and more costly than SoC alone, resulting in an ICER equal to €9,986 per QALY gained indicating that ivabradine plus SoC was a cost-effective alternative (Table 3).

**Table 3:** **Deterministic cost - effectiveness results of Base Case Lifetime Analysis**

	**Ivabradine + Standard therapy**	**Standard therapy**	**Incremental**
**Costs**			
Total costs (€)	8,655	5,873	2,792
Drug acquisition cost (€)	5,340	2,374	2,966
Hospitalization costs (€)	1,833	1,781	52
HF management costs (€)	1,492	1,754	−262
**Health outcomes**			
QALYs	4.27	3.99	0.28
LYs	5.86	5.61	0.25
**Incremental analysis**			
ICER per QALY (€)			9,986
ICER per LY (€)			11,002

ICER: incremental-cost-effectiveness ratio; LY: life-year; QALY: quality-adjusted-life-year.

### One way sensitivity analysis

#### Parameter sensitivity analysis

**Figure 2:**
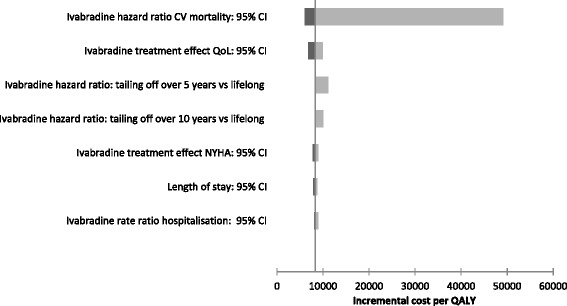
**Tornado diagram: results from one way sensitivity analysis.**

The results of the sensitivity analysis conducted on parameter estimates are reported in the Tornado diagram featured in Figure 2. It should be noted that the Tornado diagram has been estimated by applying average covariate values into the risk equations for simplicity (base case ICER estimate derived using this method €9,196 – approximately €790 less than the base case ICER estimate - €9,986 - which was derived by applying individual patient profiles into the risk equations one at a time). The results of sensitivity analysis indicated that the ICER was likely to remain below the threshold of €36,000 per QALY gained in all scenarios, with the exception of the ivabradine HR for CV mortality where the ICER exceeded €36,000 when the HR was set at the upper limit of its 95% confidence interval. The rest of findings of this analysis suggested a robustness of results to clinically plausible changes in assumptions.

#### Structural sensitivity analysis

The results of the structural sensitivity analysis are reported in Table 4. The structural scenario analyses indicate that assumptions have generally been conservative with respect to the ICER. The use of alternative parametric distributions (exponential and Weibull) for CV mortality estimation also both resulted in a more favorable ICER estimate, while the use of Kaplan Meier data of SHIFT study to estimate CV mortality resulted in a higher ICER still below the threshold of €36,000 per QALY gained. Moreover, structural sensitivity analysis showed that when the treatment effect of ivabradine was modeled only on HF mortality, with other CV death modeled as equivalent to SoC, a more favorable ICER estimate was achieved (€7,903/QALY gained). Additionally, the results were insensitive to the consideration of titration visit cost and ECG cost in the model as well as to changes in utility estimates to other data source (SHIFT predicted versus external literature) or inclusion of an age-adjustment (higher utility loss as the modeled cohort aged).

**Table 4:** **Summary of structural sensitivity analysis**

**Parameter**	**Min ICER**	**Max ICER**	**Output differences**
Treatment effect: HF only endpoints	€7,903	€9,196	€1,293
CV: mortality Kaplan Meier vs Gompertz	€9,196	€10,313	€1,117
Ivabradine treatment duration: 5 years vs lifelong	€8,145	€9,196	€1,051
NYHA extrapolation: LoCF vs Assumption based	€9,099	€9,669	€569
QoL weights: Exclude age adjustment vs include age adjustment	€9,099	€9,353	€254
CV mortality: Weibull vs Gompertz	€8,946	€9,196	€250
QoL weights: SHIFT predicted vs external literature	€8,859	€9,099	€240
NYHA extrapolation: LoCF vs SHIFT predicted	€8,966	€9,099	€133
CV mortality: exponential vs gompertz	€9,070	€9,196	€126
Titration visit and ECG costs excluded vs included	€9,077	€9,099	€22

CV: cardiovascular; QoL: Quality of Life; ICER: incremental cost effectiveness ratio; HF: Heart Failure; ECG: electrocardiographic changes; NYHA: New York Heart Association; LoCF: Last observation carried forward.

#### Subgroup analyses

The ICER values have been estimated for a series of subgroup populations. These subgroup populations were designed with reference to the clinical trial protocol and previous SHIFT study populations. The subgroup analyses indicated that the use of ivabradine plus SoC seems to be more cost-effective alternative over SoC alone among patients with more severe heart failure (NYHA class III and IV), as the ICER becomes lower when the severity of heart failure increases. The results of subgroup analyses are presented in Table 5. It should be noted that ivabradine plus SoC remains a cost effective alternative in all subgroups.

**Table 5:** **Results from subgroup analyses**

**Subgroup**	**Standard care**		**Ivabradine + Standard care**		**Incremental costs & outcomes**				
	**Total costs (in €)**	**Total QALYs**	**Total costs (in €)**	**Total QALYs**	**Costs (in €)**	**LYs**	**QALYs**	**Cost/LY (in €)**	**Cost/QALY (in €)**
All patients (HR ≥ 75 b.p.m.)	5,873	3.99	8,665	4.27	2,792	0.25	0.28	11,002	9,986
Age < 75 years	6,033	4.14	8,932	4.43	2,899	0.27	0.29	10,897	9,909
Age ≥ 75 years	4,350	2.54	6,123	2.69	1,772	0.14	0.16	12,944	11,355
NYHA II	6,358	4.55	9,498	4.84	3,140	0.24	0.28	12,935	11,112
NYHA III	5,528	3.55	8,059	3.83	2,531	0.27	0.28	9,542	9,044
NYHA IV	3,383	1.79	4,726	2.00	1,343	0.22	0.21	6,018	6,445
HF duration <0.6 years	6,683	5.02	10,063	5.35	3,379	0.28	0.33	12,085	10,287
HF duration ≥0.6 < 2 years	5,881	4.10	8,752	4.39	2,871	0.26	0.29	10,919	9,883
HF duration ≥2 < 4.8 years	5,489	3.66	8,138	3.92	2,648	0.24	0.26	10,916	10,168
HF duration ≥4.8 years	5,466	3.20	7,755	3.44	2,290	0.23	0.24	9,931	9,527
No beta blocker	5,144	3.08	7,384	3.39	2,240	0.31	0.31	7,239	7,267
Beta blocker < half target dose	5,481	3.60	7,992	3.87	2,511	0.25	0.27	9,942	9,250
Beta blocker = > half target dose < target dose	6,338	4.45	9,438	4.73	3,100	0.24	0.28	12,794	11,115
Beta blocker = > target dose	6,440	4.64	9,684	4.92	3,244	0.24	0.28	13,602	11,619
LVEF < 26%	5,407	3.31	7,709	3.60	2,302	0.28	0.29	8,356	8,072
LVEF ≥26% < 30%	5,483	3.66	8,052	3.94	2,569	0.25	0.27	10,163	9,440
LVEF ≥30 < 33%	5,993	4.19	8,947	4.47	2,955	0.26	0.28	11,558	10,400
LVEF ≥ 33%	6,443	4.64	9,688	4.92	3,245	0.23	0.27	13,977	11,807
Non-diabetic	5,746	4.04	8,577	4.32	2,831	0.25	0.28	11,210	10,112
Diabetic	6,150	3.86	8,859	4.14	2,709	0.26	0.28	10,556	9,710
No prior CAD	5,839	4.20	8,719	4.52	2,880	0.29	0.32	9,907	9,051
Prior CAD	5,887	3.90	8,643	4.16	2,756	0.24	0.26	11,549	10,448

HR: Heart rate; CAD: coronary artery disease; HF: Heart Failure; LY: life Years; QALY: Quality-adjusted-life year; NYHA: New York Heart Association; LVEF: Left Ventricular Ejection Fraction.

#### Probabilistic sensitivity analysis

**Figure 3:**
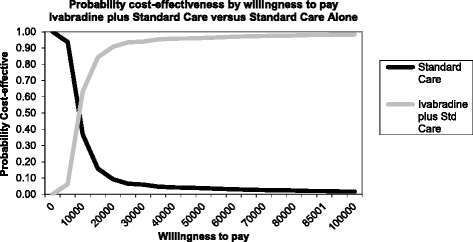
**The acceptability curve for lifetime analysis.**

In the base case analysis, probabilistic analysis confirms the deterministic results. In particular, the analysis showed that ivabradine plus SoC is the more cost-effective comparator (assuming a willingness to pay threshold of €36,000) for the majority of the run. The probabilistic sensitivity analysis showed that the likelihood of Ivabradine plus SoC being cost-effective at a willingness-to-pay threshold of €36,000/QALY was found to be 95% compared with SoC alone. The CEAC confirms that a willingness-to-pay threshold of €36,000 ivabradine plus SoC was the regimen of choice (Figure 3).

## Discussion

In the present study, a Markov modeling approach was used to evaluate the cost-effectiveness of ivabradine in a lifetime horizon for CHF patients with a baseline heart rate ≥ 75 b.p.m., compared with current standard of care, in Greece. The analysis revealed that ivabradine plus SoC appeared to be more effective and more costly compared to SoC, with an ICER well below the threshold of €36,000 per QALY gained, indicating that ivabradine is a cost-effective alternative for the management of CHF in Greece.

Our results are consistent with those submitted to NICE, in which ivabradine plus SoC was found to be associated with an increased cost of £8,498 per QALY gained compared with SoC. The ICER was sensitive to changes in the treatment effects of ivabradine at the upper bound 95% confidence interval for CV mortality (HR: 0.80-1.03). However, the risk equations were developed using data from the total SHIFT population (heart rate ≥70 bpm). Therefore, this scenario analysis overestimates the upper bound hazard ratio and ICER estimate for the licensed indication (heart rate ≥75 bpm) [11].

Although the methodology adopted followed the standard recommendations and various sensitivity analyses were conducted to fully explore uncertainty, the analysis cannot substitute for real-life direct comparisons amongst the alternative treatments. Hence, post-launch observational studies are needed to verify the conclusions obtained from analyses such as the present. A more complete analysis from a broader (societal) perspective may also be worthwhile. True health care and patient direct and indirect costs are higher than those considered in the present analysis, and therefore the cost-effectiveness of a new therapy may be more favourable from a societal perspective. Moreover, in the present analysis it was assumed that the clinical outcomes obtained from the SHIFT trial were applicable to the Greek health care setting. The use of data derived from clinical trials may be questionable, however given the lack of locally generated data, this was the only relevant source for clinical data; one may argue that pivotal trials are almost universally used to build models for pricing and reimbursement decisions. Moreover, only two local experts and not an expert panel was used to obtain local resource utilization and validate some of the assumptions considered in the model. This fact might raise concerns about the subjectivity of model inputs and leave space for challenging the study results. Nonetheless, he is a well-known cardiologist with extensive clinical experience on management of CHF, in Greece. Finally, it should be noted that the results have to be considered in the strict Greek setting and on the basis of the present time resource and drug prices. If any of the underlying parameters change, so may the results and the conclusions of the analysis.

## Conclusions

We can conclude that the use of Ivabradine plus SoC therapy may be regarded as a cost effective alternative when compared with SoCpy alone.

## Additional files

Additional file 1:
**Health care utilization and drug cost.**
Additional file 2:
**Proportion of patients using each standard therapy and mean drug daily dose.**

## Abbreviations

CHF: Chronic heart failure
SoC: Standard care
HF: Heart failure
NYHA: New York Heart Association
HR-QoL: health-related quality of life
QoL: Quality of life
ACE: angiotensin-converting-enzyme
ARBs: angiotensin receptors blockers
CV: Cardiovascular
HR: Hazard ratio
CI: Confidence interval
NICE: National Institute for Health and Care Excellence
LoS: Length of stay
ICU: Intensive care unit
DRGs: Diagnostic related groups
GDP: Gross domestic product
QALY: Quality adjusted life year
LY: Life year
ICER: Incremental cost effectiveness ratio
CEACs: Cost-effectiveness acceptability curves
ECG: Electrocardiographic changes
LVEF: Left ventricular ejection fraction
LoCF: Last observation carried forward
