# Supplementary material for: Economic evaluation of ivabradine in the treatment of chronic heart failure in Greece
Source: BMC Health Serv Res. 2014 Dec 11;14:631. doi: 10.1186/s12913-014-0631-0 (PMC4269870; doi:10.1186/s12913-014-0631-0)
Supplement: Additional file 1: — Health care utilization and drug cost. [file 12913_2014_631_MOESM1_ESM.docx]

**Additional file 1**

Health Care Utilization and Drug Cost

| Description | Patient (%) | Frequency | | Weighted Cost |
| --- | --- | --- | --- | --- |
| Physician Visit | 100 % | 1 per 2 month | | - |
| Blood Tests | 100 % | 1 per 2 month | | 1.20 € |
| Biochemical Tests | 100 % | 1 per 2 month | | 6.16 € |
| BNP & ECG | 100 % | 1 per 2 month | | 3.44 € |
| Echocardiogram | 100 % | 1 per 6 month | | 9.91 € |
| Holter Monitoring Test | 80 %  20 % | 1 per 6 month  1 per 3 month | | 2.10 € |
| Cardio Pulmonary Test | 60 % | 1 per 12 month | | 1.87 € |
| MRI cardiac | 10 % | 1 per 12 month | | 1.37 € |
|  |  | |  | |
| Description | **Proportion of the cohort (%)** | | **Total Cost per Month (€)** | |
| Ace inhibitors | 0.80 % | | 6.461 € | |
| Angiotensin receptor blockers | 0.10 % | | 1.028 € | |
| Aldosterone | 0.50 % | | 8.021 € | |
| Digitalis | 0.30 % | | 0.019 € | |
| Loop diuretics | 0.74 % | | 0.641 € | |
| Beta Blockers | 0.80 % | | 3.369 € | |
| Statins | 0.61 % | | 9.349 € | |
| Antiarrhythmics | 0.14 % | | 0.510 € | |
| Anticoagulants | 0.12 % | | 1.862 € | |
| Anticoagulants | 0.16 % | | 0.302 € | |
| Nitrates | 0.35 % | | 2.677 € | |
| Anti-Ishaemic | 0.14 % | | 0.857 € | |
