# Supplementary material for: Economic evaluation of ivabradine in the treatment of chronic heart failure in Greece
Source: BMC Health Serv Res. 2014 Dec 11;14:631. doi: 10.1186/s12913-014-0631-0 (PMC4269870; doi:10.1186/s12913-014-0631-0)
Supplement: Additional file 2: — Proportion of patients using each standard therapy and mean drug daily dose. [file 12913_2014_631_MOESM2_ESM.docx]

Additional file 2

Proportion of patients using each standard therapy and mean drug daily dose

| **Drug type** | **Most commonly prescribed** | **Description** | **Proportion of cohort** | **Mean dosage per day mg** | **Price per mg**** | **Total cost per month** |
| --- | --- | --- | --- | --- | --- | --- |
| **Ace inhibitors** | **Combined** |  | **0.80** | **10.50** | 0.0350 | 6.461 |
|  | Ramipril | TRIATEC 5MG/TAB BTx20 (BLIST 2x10) | 0.40 | 5.00 | 0.0574 | 3.495 |
|  | Enalapril | RENITEC 20MG/TAB BTx10 (BLIST 1x10) | 0.30 | 20.00 | 0.0158 | 2.879 |
|  | Captopril | CAPOTEN 25MG/TAB BTX14(BLISTER 1x14) | 0.10 | 5.00 | 0.0060 | 0.091 |
|  | perindopril | COVERSYL 5MG/TAB BTx30 | 0.10 | 5.00 | 0.0469 | 0.714 |
|  | qouinapril | ACCUPRON 5MG/TAB BTX28(BLISTERS) | 0.05 | 20.00 | 0.0183 | 0.557 |
|  | lisinopril | ZESTRIL 10MG/TAB BTx14(BLIST1x14) | 0.05 | 10.00 | 0.0223 | 0.340 |
| **Angiotensin receptor blockers** | **Combined** |  | **0.10** | **23.20** | **0.0203** | **1.028** |
|  | Valsartan | DIOVAN 160MG/TAB BTx28 (BLIST1x28) | 0.05 | 160.00 | 0.0017 | 0.419 |
|  | candesartan | ATACAND 16MG/ΤΑΒ BTx28(BLIST2x14) | 0.95 | 16.00 | 0.0213 | 9.861 |
| **Aldosterone** | **combined** |  | **0.50** | **25.00** | **0.0211** | **8.021** |
|  | spironolactone | ALDACTONE 25MG/TAB ΒT x 20 | 0.50 | 25.00 | 0.0031 | 1.172 |
|  | eplerenone | INSPRA 25MG/TAB BT x 20 | 0.50 | 25.00 | 0.0391 | 14.870 |
| **Digitalis** | **Digoxin** | **DIGOXIN TABL BT 25X0.25MG** | **0.30** | **0.13** | **0.160** | **0.0190** |
| **Loop diuretics** | **Furosemide** | **FUROSEMIDE/FRESENIUS KABI 20MG/2ML BT x 5 AMP x 2 ML** | **0.74** | **59.36** | **0.0005** | **0.6418** |
| **Beta blockers** | **combined** |  | **0.80** | **15.50** | **0.0127** | **3.369** |
|  | carvedilol | CARVEPEN 12,5MG/TAB BTx28 (BLISTER 2x14) | 0.80 | 12.50 | 0.0119 | 3.630 |
|  | metoprolol | LOPRESOR 100MG/TAB ΒΤΧ40(ΣΕBLISTERS) | 0.10 | 50.00 | 0.0007 | 0.106 |
|  | nebivolol | LOBIVON 5MG/TAB BTx28 (BLIST 2x14) | 0.10 | 5.00 | 0.0312 | 0.475 |
| **Statins** | **Simvastatin** | **ZOCOR 20MG/TAB BT x 10** | **0.61** | **23.39** | **0.0215** | **9.349** |
| **Antiarrhythmics** | **Amiodarone** | **ANGORON TABL BT 30x200 MG** | **0.14** | **200.00** | **0.0006** | **0.510** |
| **Anticoagulants** | **Clopidogrel** | **CLOPIDOGREL APOTEX 75MG/TAB BTx 28 σε BLISTERS** | **0.12** | **74.71** | **0.0068** | **1.862** |
| **Anticoagulants** | **Acenocoumarol** | **SINTROM 4MG/TAB ΒΤΧ20(BLIST 2X10 )** | **0.16** | **3.06** | **0.0202** | **0.302** |
| **Nitrates** | **Isosorbide mononitrate** | **MONOSORDIL 60MG/CAP BTx14(BLIST 2 x 7)** | **0.35** | **53.24** | **0.0055** | **2.677** |
| **Anti-ischaemic** | **Trimetazidine** | **VASTAREL 20MG/TAB BTx60 (BLIST 4x15)** | **0.14** | **63.30** | **0.0044** | **0.857** |
|  | **Ivabradine** | **PROCORALAN 2,5 MG/TAB *** | **0.10** | **5.00** | **0.280** | **21.28** |
|  |  | **PROCORALAN 5 MG/TAB BT X 56 σε ΒLISTERS** | **0.70** | **10.00** | **0.140** | **42.56** |
|  |  | **PROCORALAN 7,5 MG/TAB BT X 56 σε ΒLISTERS** | **0.20** | **15.00** | **0.090** | **43.01** |

*not available in Greece, the unit cost of this was calculated based on the PROCORALAN 5 MG/TAB BT X 56 σε ΒLISTERS
